# The impact of COVID-19 pandemic on well-being of Italian physicians: a report from the Italian Society of Internal Medicine (SIMI) national survey

**DOI:** 10.1007/s11739-022-03108-4

**Published:** 2022-10-01

**Authors:** Giulio Francesco Romiti, Leonardo Bencivenga, Rosanna Villani, Sebastiano Cicco, Antonio Cimellaro, Andrea Dalbeni, Giovanni Talerico, Antonello Pietrangelo, Giorgio Sesti, Vincenzo Zaccone, on behalf of Giovani Internisti SIMI (GIS), Giuseppe Armentaro, Maria Immacolata Arnone, Milena Barone, Leonardo Bencivenga, Lorenzo Bertolino, Sara Bianco, Nicolò Binello, Simona Brancati, Elena Buzzetti, William Capeci, Sebastiano Cicco, William Cordeddu, Rosa Curcio, Andrea Dalbeni, Marco D’Abbondanza, Salvatore D’Agnano, Damiano D’Ardes, Martina De Feo, Salvatore Di Marca, Emilia Donnarumma, Marco Fei, Emanuele Filippini, Carmine Gabriele Gambino, Rosa Lombardi, Alberto Maria Marra, Massimo Mattioli, Giuseppe Miceli, Silvia Noviello, Gaia Olivieri, Donatella Padula, Roberta Parente, Chiara Pes, Salvatore Piano, Francesca Serena Pignataro, Sonia Poma, Enrica Porceddu, Marco Ricchio, Giulio Francesco Romiti, Anna Sabena, Marco Salice, Andrea Salzano, Moris Sangineto, Ambra Savona, Caterina Savrié, Mario Stabile, Nicola Susca, Giovanni Talerico, Thomas Teatini, Elisabetta Tombolini, Matteo Traversa, Elia Vettore, Alessandro Vignali, Rosanna Villani, Luca Vilardi, Vincenzo Zaccone

**Affiliations:** 1 Department of Translational and Precision Medicine, Sapienza – University of Rome, Rome, Italy; 2 Dipartimento Di Scienze Biomediche Avanzate, Università Degli Studi Di Napoli “Federico II”, Naples, Italy; 3 Gérontopôle de Toulouse, Institut du Vieillissement, CHU de Toulouse, Toulouse, France; 4 Liver Unit - Dipartimento Di Scienze Mediche E Chirurgiche, Università Degli Studi Di Foggia, Foggia, Italy; 5 Unit of Internal Medicine “Guido Baccelli”, Department of Biomedical Sciences and Human Oncology, University of Bari, Piazza Giulio Cesare, 11, 70124 Bari, Italy; 6 UOC Medicina Generale - Azienda Ospedaliera “Pugliese Ciaccio”, Catanzaro, Italy; 7 Sezione Di Medicina Interna C E Liver Unit, Dipartimento Di Medicina, Azienda Ospedaliero Universitaria Di Verona, Verona, Italy; 8 UOC Medicina Interna - Policlinico Casilino, Rome, Italy; 9 Department of Surgical and Medical Sciences for Children and Adults, Internal Medicine Unit, University of Modena and Reggio Emilia, Modena, Italy; 10 Department of Clinical and Molecular Medicine, Sapienza University, Rome, Italy; 11 Medicina Interna Generale E Subintensiva - Azienda Ospedaliera Universitaria Ospedali Riuniti, Ancona, Italy

**Keywords:** COVID-19, Healthcare, Well-being, SARS-CoV-2, Work-related stress

## Abstract

Over the past few years, COVID-19 pandemic has imposed a high toll worldwide, with a high burden of morbidity and mortality. Healthcare practitioners (HCPs) have been in the frontline since the beginning of the outbreak, and the high level of stress have affected their physical and mental status, as well as their relationships. We aimed at exploring the self-reported changes in comprehensive well-being in a cohort of Italian physicians. An online-based survey was administered to the members of the Italian Society of Internal Medicine (SIMI) between March and June 2021. The survey was based on 32 multiple-choice questions exploring self-reported physical and mental well-being, as well as changes in workloads, work-related feelings and physicians’ relationship with patients, colleagues and families. 228 physicians (mean age: 35.7 ± 9.8 years) participated in the survey; 120 (52.6%) were residents, 196 (86.0%) worked in COVID-19 units and 65 (28.5%) had COVID-19 during the pandemic. A significant proportion of respondents reported to have experience onset or worsening of physical and mental symptoms, with insomnia/sleep disorders (58.3%) and mood swings (47.8%) being the most common, respectively. The burden of physical and mental consequences was broadly higher among residents compared to specialists, with the former reporting more frequently an increase in the number of worked hours (*p* = 0.020) and being more frequently infected with COVID-19 (35.0% vs. 21.3, *p* = 0.032). Moreover, familiar and doctor–patient relationships were also considerably affected. Physicians have been suffering a wide spectrum of physical, mental and relational consequences during COVID-19 pandemic, with youngest doctors being more likely to present several physical and mental health symptoms. Further studies are needed to evaluate long-term consequences of COVID-19 pandemic on the well-being of HCPs, and potential preventive strategies.

**Supplementary Information:**

The online version contains supplementary material available at 10.1007/s11739-022-03108-4.

## Introduction

The Coronavirus Disease-19 (COVID-19) has imposed a significant burden of morbidity and mortality worldwide, with millions of deaths and an unprecedented pressure on national health systems and healthcare providers (HCP). Italy was the second country that experienced a large spread of SARS-CoV-2 virus, resulting in direct and indirect effects on the National Healthcare system [1, 2]. Caught in the middle, between uncertainties and overwhelming workloads [3], HCPs experienced a significant amount of stress, with significant (although often neglected) impact on their physical and psychological status.

Several previous evidence have already described the toll of the pandemic on the risk of burnout and psychological consequences among HCPs [4, 5]. Particularly, the risk of burnout syndrome—which is characterized by emotional exhaustion, depersonalization and reduced personal accomplishment in individuals professionally involved with others [6] – has been repeatedly described. Burnout syndrome, indeed, is increasingly found among HCPs [7, 8].

Moreover, the impact of COVID-19 pandemic on psychological well-being of physicians and HCPs has also been described as multifaceted and complex [9, 10]. Several factors may represent sources of psychological stress for HCPs, including the abrupt increase in the workload, the unexpected loss of colleagues and family members, traumatic experiences in the patient–physicians relationship, as well as the great uncertainty related to the treatment and management of COVID-19 patients; finally, personal history of having had COVID-19 may also play a role. All these factors have generated an unprecedented scenario with severe detrimental effects on the HCPs well-being, both from a physical and psychological point of view.

These negative effects may have specifically impacted younger HCPs, or those at an early career stage [11]. Trainee and residents have experienced a significant cost, due to the impact of the pandemic on learning programs and clinical rotations, as well as rescheduling of clinical activities, which has often placed trainee in a suddenly central role in the care of COVID-19 patients. Unsurprisingly, some reports have already showed how the COVID-19 pandemic exerted a detrimental effect on the overall and psychological well-being of trainee physicians [12–14]. Finally, regional differences in the burden of COVID-19 cases, particularly during the first phase of the outbreak [15], may have played a role in shaping the impact of the pandemic on HCPs well-being.

In this study, we report the results of a nationwide survey aimed at evaluating the impact of COVID-19 pandemic on the physical and psychological well-being of Italian physicians. We also appraised changes in work-related environments and interactions, as well as the impact of the relationships between physicians and their patients and families. Finally, we assessed the effects in different group of physicians according to their job role, the involvement in COVID-19 units, and having experienced a SARS-CoV-2 infection. Regional differences were also explored.

## Methods

Data were collected from a web-based survey of physicians who are members of the Italian Society of Internal Medicine (*Società Italiana di Medicina Interna,* SIMI). The survey was conducted between March 1st, 2021 and June 30th, 2021. The questionnaire-based survey was drafted in Italian and included 32 multiple-choice questions that aimed to explore the self-reported effects of COVID-19 pandemic on several domains, including personal life, work status and mental health.

Each member of SIMI was asked to compile the survey via an e-mail based invitation that contained a direct link to the questionnaire.

Data were collected in an anonymised form. Each respondent was assigned a unique identification number and no personal information was collected or recorded.

The study was performed in accordance with the principles of the Declaration of Helsinki, and was exempted by Ethic Committee approval because of the anonymous nature of the survey. Participants provided their online written informed consent before filling in the survey. The study was approved by SIMI and the Independent Research Centre of SIMI (*Centro Ricerca Indipendente della SIMI,* CRIS).

### Questionnaire and comparisons

Questions and responses of the questionnaire are reported in Table 1 and Table 2. Briefly, the survey explored different domains of the impact of the COVID-19 pandemic on the respondents’ well-being. Participants were asked about their job position and activities, as well as the changes experienced during the pandemic compared to six months before the COVID-19 outbreak. Work effort was assessed as the number of night shifts, and weekly hours spent at work. Respondents were also asked about the number of COVID-19 patients treated, and the personal COVID-19 infection status during the outbreak.

**Table 1:** Baseline characteristics and physical and mental symptoms of respondents

Variables, *n* (%)	All respondents (*n* = 228)	Specialists (*n* = 108)	Residents (*n* = 120)	*P*	Not worked in COVID-19 units (*n* = 32)	Worked in COVID-19 units (*n* = 196)	*P*	Not had COVID-19 (*n* = 163)	Had COVID-19 (*n* = 65)	*P*
Age, years (Mean ± SD)	35.7 ± 9.8	42.6 ± 10.3	29.5 ± 2.6	< 0.001	37.5 ± 10.9	35.4 ± 9.6	0.275	36.4 ± 10.1	34.1 ± 8.8	0.119
Job role										
Resident	120 (52.6)	–	–		18 (56.2)	102 (52.0)	0.802	78 (47.9)	42 (64.6)	0.032
Specialist (Consultant, Attending)	108 (47.4)	–	–		14 (43.8)	94 (48.0)		85 (52.1)	23 (35.4)	
Worked in COVID-19 wards				0.802						0.493
Yes	196 (86.0)	94 (87.0)	102 (85.0)		–	–		138 (84.7)	58 (89.2)	
No	32 (14.0)	14 (13.0)	18 (15.0)		–	–		25 (15.3)	7 (10.8)	
History of COVID-19				0.032			0.493			
Yes	65 (28.5)	23 (21.3)	42 (35.0)		7 (21.9)	58 (29.6)		–	–	
No	163 (71.5)	85 (78.7)	78 (65.0)		25 (78.1)	138 (70.4)		–	–	
Medical specialty				0.504			0.006			0.747
Internal medicine	161 (70.6)	75 (69.4)	86 (71.7)		19 (59.4)	142 (72.4)		114 (69.9)	47 (72.3)	
Geriatrics	23 (10.1)	13 (12.0)	10 (8.3)		1 (3.1)	22 (11.2)		15 (9.2)	8 (12.3)	
Emergency medicine	5 (2.2)	1 (0.9)	4 (3.3)		0 (0.0)	5 (2.6)		4 (2.5)	1 (1.5)	
Other	39 (17.1)	19 (17.6)	20 (16.7)		12 (37.5)	27 (13.8)		30 (18.4)	9 (13.8)	
Workload										
More than 45 h per week	80 (35.1)	29 (26.9)	51 (42.5)	0.020	8 (25.0)	72 (36.7)	0.276	50 (30.7)	30 (46.2)	0.040
More than 4 night shifts per month	65 (28.5)	38 (35.2)	27 (22.5)	0.049	5 (15.6)	60 (30.6)	0.126	52 (31.9)	13 (20.0)	0.102
Questionnaire—physical and mental symptoms										
Did you notice the onset or worsening of any of these physical signs/symptoms?										
Esophageal reflux, heartburn	69 (30.3)	34 (31.5)	35 (29.2)	0.814	24 (75.0)	135 (68.9)	0.623	42 (25.8)	27 (41.5)	0.029
Asthenia	68 (29.8)	36 (33.3)	32 (26.7)	0.340	9 (28.1)	59 (30.1)	0.985	45 (27.6)	23 (35.4)	0.318
Unintentional weight changes	81 (35.5)	38 (35.2)	43 (35.8)	1.000	10 (31.2)	71 (36.2)	0.729	59 (36.2)	22 (33.8)	0.856
Headache	83 (36.4)	40 (37.0)	43 (35.8)	0.960	10 (31.2)	73 (37.2)	0.649	56 (34.4)	27 (41.5)	0.387
Dyspepsia	23 (10.1)	12 (11.1)	11 (9.2)	0.790	28 (87.5)	177 (90.3)	0.863	12 (7.4)	11 (16.9)	0.055
Abdominal pain/bowel habit changes	46 (20.2)	25 (23.1)	21 (17.5)	0.370	3 (9.4)	43 (21.9)	0.160	29 (17.8)	17 (26.2)	0.216
Muscular pain/tremor	19 (8.3)	11 (10.2)	8 (6.7)	0.472	0 (0.0)	19 (9.7)	0.135	12 (7.4)	7 (10.8)	0.565
Insomnia/sleep disorder	133 (58.3)	61 (56.5)	72 (60.0)	0.687	16 (50.0)	117 (59.7)	0.402	92 (56.4)	41 (63.1)	0.442
Did you notice the onset or worsening of any of these other sign/symptoms?										
Anxiety/panic attack	56 (24.6)	24 (22.2)	32 (26.7)	0.532	8 (28.1)	47 (24.0)	0.777	39 (23.9)	17 (26.2)	0.855
Apathy	68 (29.8)	20 (18.5)	48 (40.0)	0.001	9 (28.1)	59 (30.1)	0.985	41 (25.2)	27 (41.5)	0.023
Amnesia	21 (9.2)	10 (9.3)	11 (9.2)	1.000	5 (15.6)	16 (8.2)	0.306	10 (6.1)	11 (16.9)	0.022
Crying fit	51 (22.4)	18 (16.7)	33 (27.5)	0.072	7 (21.9)	44 (22.4)	1.000	35 (21.5)	16 (24.6)	0.735
Attention deficits/difficulties in concentrating	82 (36.0)	31 (28.7)	51 (42.5)	0.042	13 (40.6)	69 (35.2)	0.694	52 (31.9)	30 (46.2)	0.061
Eating behaviour disorders	52 (22.8)	29 (26.9)	23 (19.2)	0.221	4 (12.5)	48 (24.5)	0.204	40 (24.5)	12 (18.5)	0.416
Depression	35 (15.4)	16 (14.8)	19 (15.8)	0.977	5 (15.6)	30 (15.3)	1.000	24 (14.7)	11 (16.9)	0.832
Mood changes	109 (47.8)	49 (45.4)	60 (50.0)	0.571	12 (37.5)	97 (49.5)	0.285	71 (43.6)	38 (58.5)	0.059

**Table 2:** Work-related symptoms and self-reported familiar impact in respondents

Variables, *n* (%)		All respondents (*n* = 228)	Specialists (*n* = 108)	Residents (*n* = 120)	*P*	Not worked in COVID-19 wards (*n* = 32)	Worked in COVID-19 wards (*n* = 196)	P	Not had COVID-19 (*n* = 163)	Had COVID-19 (*n* = 65)	*P*
Questionnaire—work-related and self-reported familiar impact											
Do you think your work organization has worsened during COVID-19?											
Not worsened/improved		60 (26.3)	27 (25.0)	33 (27.5)	0.661	4 (12.5)	56 (28.6)	0.020	45 (27.6)	15 (23.1)	0.767
Slightly worsened		42 (18.4)	18 (16.7)	24 (20.0)		11 (34.4)	31 (15.8)		29 (17.8)	13 (20.0)	
Significantly worsened		126 (55.3)	63 (58.3)	63 (52.5)		17 (53.1)	109 (55.6)		89 (54.6)	37 (56.9)	
Do you think that patient–physicians relationship has worsened during COVID-19?											
Not worsened/improved		76 (33.3)	42 (38.9)	34 (28.3)	0.221	12 (37.5)	64 (32.7)	0.670	50 (30.7)	26 (40.0)	0.396
Slightly worsened		72 (31.6)	30 (27.8)	42 (35.0)		11 (34.4)	61 (31.1)		53 (32.5)	20 (30.8)	
Significantly worsened		80 (35.1)	36 (33.3)	44 (36.7)		9 (28.1)	71 (36.2)		60 (36.8)	26 (40.0)	
Do you think that your relationship with patients’ relatives has worsened during COVID-19?											
Not worsened/improved	58 (25.4)		30 (27.8)	28 (23.3)	0.559	8 (25.0)	50 (25.5)	0.662	39 (23.9)	19 (29.2)	0.683
Slightly worsened	71 (31.1)		35 (32.4)	36 (30.0)		8 (25.0)	63 (32.1)		51 (31.3)	20 (30.8)	
Significantly worsened	99 (43.4)		43 (39.8)	56 (46.7)		16 (50.0)	83 (42.3)		73 (44.8)	26 (40.0)	
Do you think that your relationship with other colleagues has worsened during COVID-19?											
Not worsened/improved		128 (56.1)	54 (50.0)	74 (61.7)	0.037	13 (40.6)	115 (58.7)	0.161	88 (54.0)	40 (61.5)	0.569
Slightly worsened		62 (27.2)	38 (35.2)	24 (20.0)		12 (37.5)	50 (25.5)		46 (28.2)	16 (24.6)	
Significantly worsened		38 (16.7)	16 (14.8)	22 (18.3)		7 (21.9)	31 (15.8)		29 (17.8)	9 (13.8)	
Did you notice the onset or worsening of any of these when at work?											
Work-related anxiety		60 (26.3)	29 (26.9)	31 (25.8)	0.981	10 (31.2)	50 (25.5)	0.640	44 (27.0)	16 (24.6)	0.840
Amnesia		11 (4.8)	2 (1.9)	9 (7.5)	0.093	4 (12.5)	7 (3.6)	0.082	7 (4.3)	4 (6.2)	0.803
Cynicism		63 (27.6)	26 (24.1)	37 (30.8)	0.332	7 (21.9)	56 (28.6)	0.567	36 (22.1)	27 (41.5)	0.005
Attention deficits/difficulties in concentrating		70 (30.7)	33 (30.6)	37 (30.8)	1.000	12 (37.5)	58 (29.6)	0.489	45 (27.6)	25 (38.5)	0.148
Challenges in interacting with colleagues		51 (22.4)	19 (17.6)	32 (26.7)	0.138	10 (31.2)	41 (20.9)	0.284	33 (20.2)	18 (27.7)	0.297
Frustration		131 (57.5)	57 (52.8)	74 (61.7)	0.222	15 (46.9)	116 (59.2)	0.266	95 (58.3)	36 (55.4)	0.802
Fear of infecting him/herself		84 (36.8)	43 (39.8)	41 (34.2)	0.456	17 (53.1)	67 (34.2)	0.063	66 (40.5)	18 (27.7)	0.098
Low empathy		40 (17.5)	19 (17.6)	21 (17.5)	1.000	5 (15.6)	35 (17.9)	0.954	26 (16.0)	14 (21.5)	0.419
Sense of inadequacy		126 (55.3)	55 (50.9)	71 (59.2)	0.264	11 (34.4)	115 (58.7)	0.018	89 (54.6)	37 (56.9)	0.864
Sadness		85 (37.3)	37 (34.3)	48 (40.0)	0.448	11 (34.4)	74 (37.8)	0.865	62 (38.0)	23 (35.4)	0.824
Empty feelings		63 (27.6)	20 (18.5)	43 (35.8)	0.006	9 (28.1)	54 (27.6)	1.000	39 (23.9)	24 (36.9)	0.069
Want to cry		51 (22.4)	22 (20.4)	29 (24.2)	0.598	4 (12.5)	47 (24.0)	0.224	33 (20.2)	18 (27.7)	0.297
Want to quit working		78 (34.2)	39 (36.1)	39 (32.5)	0.664	11 (34.4)	67 (34.2)	1.000	54 (33.1)	24 (36.9)	0.696
After a shift in COVID-19 wards, your feelings are											
Neutral		85 (37.3)	38 (35.2)	47 (39.2)	0.717	20 (62.5)	65 (33.2)	0.005	58 (35.6)	27 (41.5)	0.229
Negative		103 (45.2)	49 (45.4)	54 (45.0)		10 (31.2)	93 (47.4)		72 (44.2)	31 (47.7)	
Positive		40 (17.5)	21 (19.4)	19 (15.8)		2 (6.2)	38 (19.4)		33 (20.2)	7 (10.8)	
During the pandemic, how many times did you think about work during your free time?											
Never/almost never		7 (3.1)	4 (3.7)	3 (2.5)	0.802	2 (6.2)	5 (2.6)	0.498	6 (3.7)	1 (1.5)	0.358
Sometimes		32 (14.0)	14 (13.0)	18 (15.0)		5 (15.6)	27 (13.8)		20 (12.3)	12 (18.5)	
Often/always		189 (82.9)	90 (83.3)	99 (82.5)		25 (78.1)	164 (83.7)		137 (84.0)	52 (80.0)	
During the COVID-19 pandemic, what issues did you experience in the patient–physician relationship?											
Impairment of verbal communication with patients		112 (49.1)	52 (48.1)	60 (50.0)	0.883	13 (40.6)	99 (50.5)	0.397	80 (49.1)	32 (49.2)	1.000
Impairment of non-verbal communication with patients		72 (31.6)	28 (25.9)	44 (36.7)	0.110	12 (37.5)	60 (30.6)	0.567	47 (28.8)	25 (38.5)	0.210
Impairment of verbal communication with patients’ relatives		118 (51.8)	54 (50.0)	64 (53.3)	0.711	20 (62.5)	98 (50.0)	0.262	88 (54.0)	30 (46.2)	0.357
Difficulties in communicating worsening prognosis or death to patients or their relatives		109 (47.8)	56 (51.9)	53 (44.2)	0.304	10 (31.2)	99 (50.5)	0.067	77 (47.2)	32 (49.2)	0.901
Difficulties in communicating/explaining the care/treatment programme		59 (25.9)	25 (23.1)	34 (28.3)	0.459	7 (21.9)	52 (26.5)	0.734	45 (27.6)	14 (21.5)	0.437
Increased mistrust by patients or their relatives		69 (30.3)	29 (26.9)	40 (33.3)	0.358	9 (28.1)	60 (30.6)	0.939	47 (28.8)	22 (33.8)	0.559
Did you experience any of the following in your familiar relationship during the pandemic?											
Apathy		32 (14.0)	16 (14.8)	16 (13.3)	0.896	1 (3.1)	31 (15.8)	0.101	20 (12.3)	12 (18.5)	0.315
Misunderstanding/arguments		58 (25.4)	24 (22.2)	34 (28.3)	0.365	10 (31.2)	48 (24.5)	0.552	31 (19.0)	27 (41.5)	0.001
Fear of infecting family members		69 (30.3)	29 (26.9)	40 (33.3)	0.358	12 (37.5)	57 (29.1)	0.451	50 (30.7)	19 (29.2)	0.956
Reduced libido/sexual activity		51 (22.4)	29 (26.9)	22 (18.3)	0.167	9 (28.1)	42 (21.4)	0.539	32 (19.6)	19 (29.2)	0.163
Sense of guilty		34 (14.9)	13 (12.0)	21 (17.5)	0.323	4 (12.5)	30 (15.3)	0.884	19 (11.7)	15 (23.1)	0.048
Sense of abandonment		28 (12.3)	14 (13.0)	14 (11.7)	0.924	1 (3.1)	27 (13.8)	0.158	17 (10.4)	11 (16.9)	0.260
Do you think that the changes in your familiar interactions have influenced your performance at work?											
No		141 (61.8)	69 (63.9)	72 (60.0)	0.824	18 (56.2)	123 (62.8)	0.765	106 (65.0)	35 (53.8)	0.292
Only slightly influenced		64 (28.1)	29 (26.9)	35 (29.2)		10 (31.2)	54 (27.6)		42 (25.8)	22 (33.8)	
Significantly influenced		23 (10.1)	10 (9.3)	13 (10.8)		4 (12.5)	19 (9.7)		15 (9.2)	8 (12.3)	

Other questions assessed the potential physical and psychological impact of the COVID-19 pandemic. Participants were asked about self-reported onset of anxious and depressive feelings, as well as having difficulty concentrating or making decisions, self-blaming, deteriorated sleep, eating disorders, tobacco and/or alcohol consumption. Finally, the impact of the pandemic on the social and familiar relationships was also assessed.

Respondents were compared according to job role (residents vs. specialists), deployment in a COVID-19 unit, and according to their personal history of COVID-19 infection. Additionally, we also compared participants based on their self-reported geographical location, i.e., physicians based in center—north of Italy (i.e., Emilia-Romagna, Friuli Venezia Giulia, Liguria, Lombardia, Marche, Piemonte, Toscana, Trentino-Alto Adige, Umbria, Valle d’Aosta and Veneto regions of Italy) vs. those from center—south of Italy (i.e., Abruzzo, Basilicata, Calabria, Campania, Lazio, Molise, Puglia, Sardegna, Sicilia).

### Statistical analysis

Continuous variables were reported according to mean ± standard deviation (SD) and median [interquartile range, IQR], and compared using Student’s *t* test or Mann–Whitney *U *test according to their normal or non-normal distribution, respectively. Categorical variables were reported as counts and percentage and compared using chi-squared test. A *p* value of < 0.05 was considered statistically significant. All analyses were performed using R 4.1.2 (R Core Team 2020, Vienna, Austria).

## Results

A total of 228 physicians (mean age: 35.7 ± 9.8 years) compiled the survey. Baseline characteristics, as well as self-reported physical and mental symptoms of respondents are reported in Table 1; Work-related symptoms and familiar impact are reported in Table 2.

Among the participants, 120 (52.6%) were residents, 108 (47.4%) attending or consulting physicians, 161 (70.6%) internal medicine specialists, 23 (10.0%) geriatricians, 5 (2.2%) physicians engaged in emergency settings, and the remaining 39 (17.1%) were other specialists. Most respondents (196, 86.0%) were directly involved in treatment and management of COVID-19 patients.

### Workplace and work-related effects

Among the respondents, 80 (35.1%) reported working more than 45 h/week, with 65 (28.5%) working more than 4 night shifts per month. Overall, 168 (73.7%) respondents reported a worsening in the organization on the workplace, with 126 (55.3%) reporting significant disruption in the work-planning.

### Physical and psychological symptoms

Overall, 190 (83.3%) participants reported at least one physical symptoms among those investigated during the COVID-19 pandemic. The most commonly reported were insomnia or sleeping difficulties (133, 58.3%), headache (83, 36.4%) and unintentional weight changes (81, 35.5%). Less common symptoms included muscle pain or tremor (19, 8.3%), dyspepsia (23, 10.1%) and abdominal pain or change in bowel habits (46, 20.2%).

On the other side, 173 (75.9%) participants reported the onset or the worsening of at least one psychological symptom. The most common symptom was mood changes (109, 47.8%), followed by loss of attention and difficulties in concentrating (82, 36.0%). Anxiety was reported by almost one out of four respondents (56, 24.6%), while a lower proportion of subjects felt depressed (35, 15.4%). Finally, 68 (29.8%) participants reported apathy, while 52 (22.8%) showed changes in eating behaviours.

During work shift, most participants reported suffering from frustration (131, 57.5%) or a sense of inadequacy (126, 55.3%), with 70 (30.7%) showing difficulties in concentrating, and 85 (37.3%) reporting sadness during shifts. A relevant proportion of participants reported fear of getting infected during shifts (84, 36.8%), and 60 (26.3%) showed sign of work-related anxiety. Consistently, 103 (45.1%) physicians reported negative feelings at the end of shift, and 189 (82.9%) reported that they spent large part of their free time thinking of work-related concerns and issues.

### Impact on relationships with patients and families

A relevant proportion of the physicians complained about worsening of the patient–physician relationship (152, 66.7%), with an even higher proportion that remarked a deteriorated interaction with patients’ relatives (170, 74.6%). Furthermore, less than a half of physicians reported worsened relationships with colleagues (100, 43.9%), while 65 (28.5%) showed an improvement.

When asked, the respondents reported that the major impairments in the patient–physician relationship were those related to the verbal communications with either patients (112, 49.1%) or their relatives (118, 51.8%); 109 (47.8%) participants reported significant troubles in communicating clinical worsening or death to the patients or their relatives. On the other side, one out of three participants (69, 30.3%) felt a higher mistrust by patients or their relatives during the pandemic.

Finally, most participants (137, 60.1%) noticed worsening of their familiar interactions and relationships. The most common determinants were the fear of infecting their family members (69, 30.3%) as well as misunderstandings and arguments (58, 25.4%). One out of five respondents reported decreased libido and reduced sexual activity (51, 22.4%), while few participants felt guilty or abandoned in respect to their family (34, 14.9% and 28, 12.3%, respectively). Although a relevant proportion (87, 38.2%) of the physicians thought that worsening of their familiar interactions had reflections on their performance at work, only 10% considered that the extent was significant.

### Comparisons between groups

**Fig. 1:**
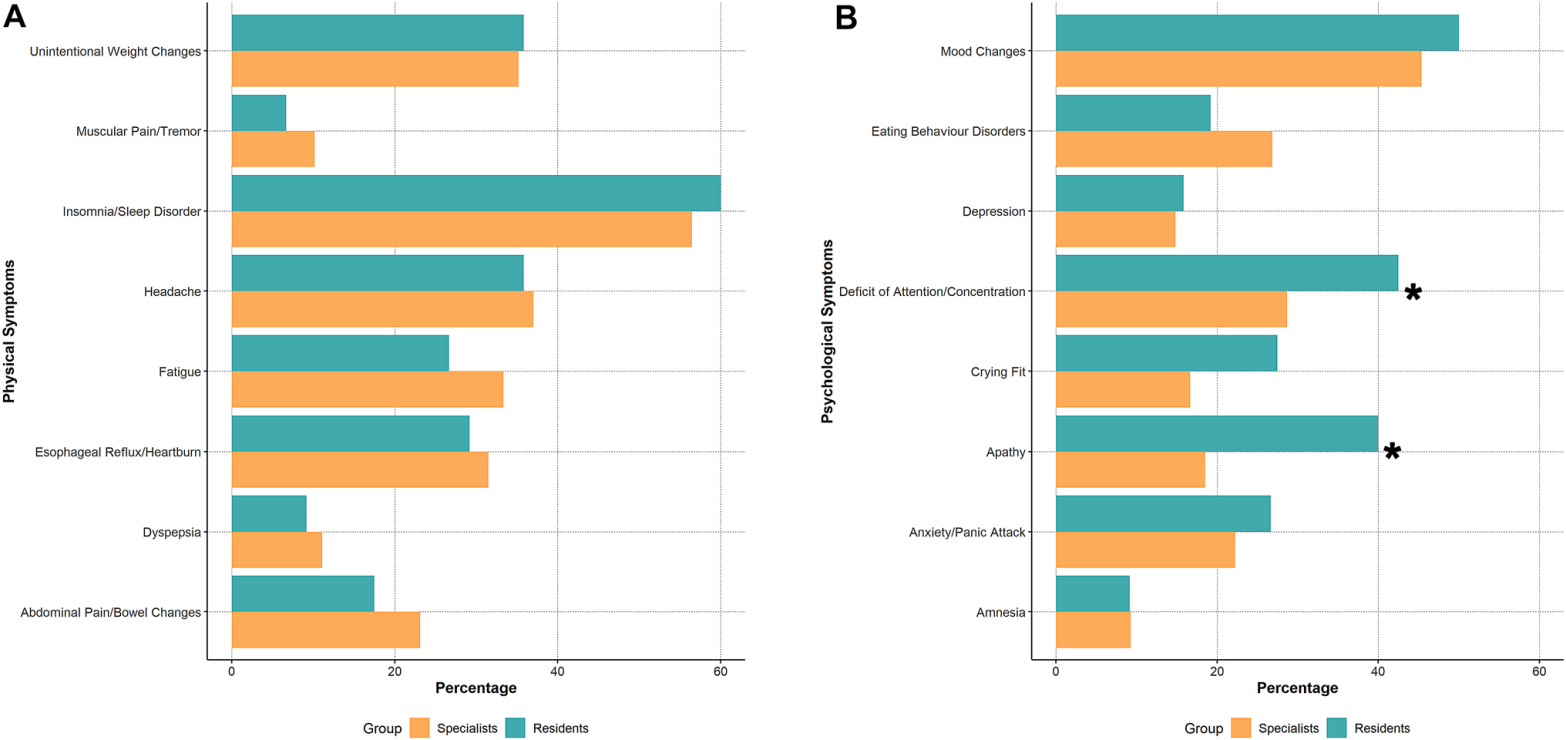
Physical and psychological symptoms reported by respondents, stratified by their job role (residents vs. specialists). Legend: * denotes significant differences at a < 0.05 *p* levels. Panel **A**: Physical symptoms; Panel **B**: Psychological symptoms

**Fig. 2:**
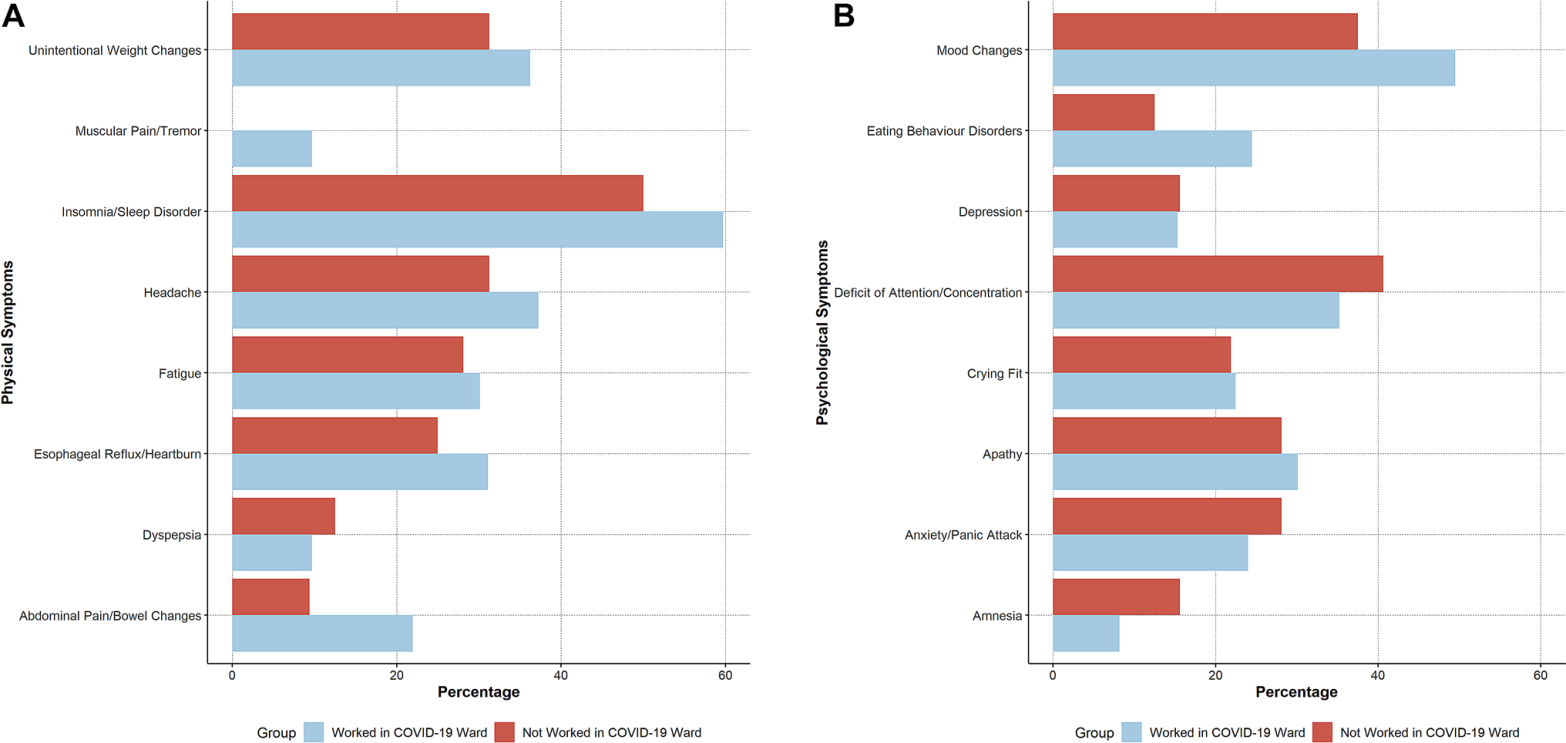
Physical and psychological symptoms reported by respondents, stratified by having or not worked in COVID-19 wards. Legend: * denotes significant differences at a < 0.05 *p* levels. Panel **A**: Physical symptoms; Panel **B**: Psychological symptoms

**Fig. 3:**
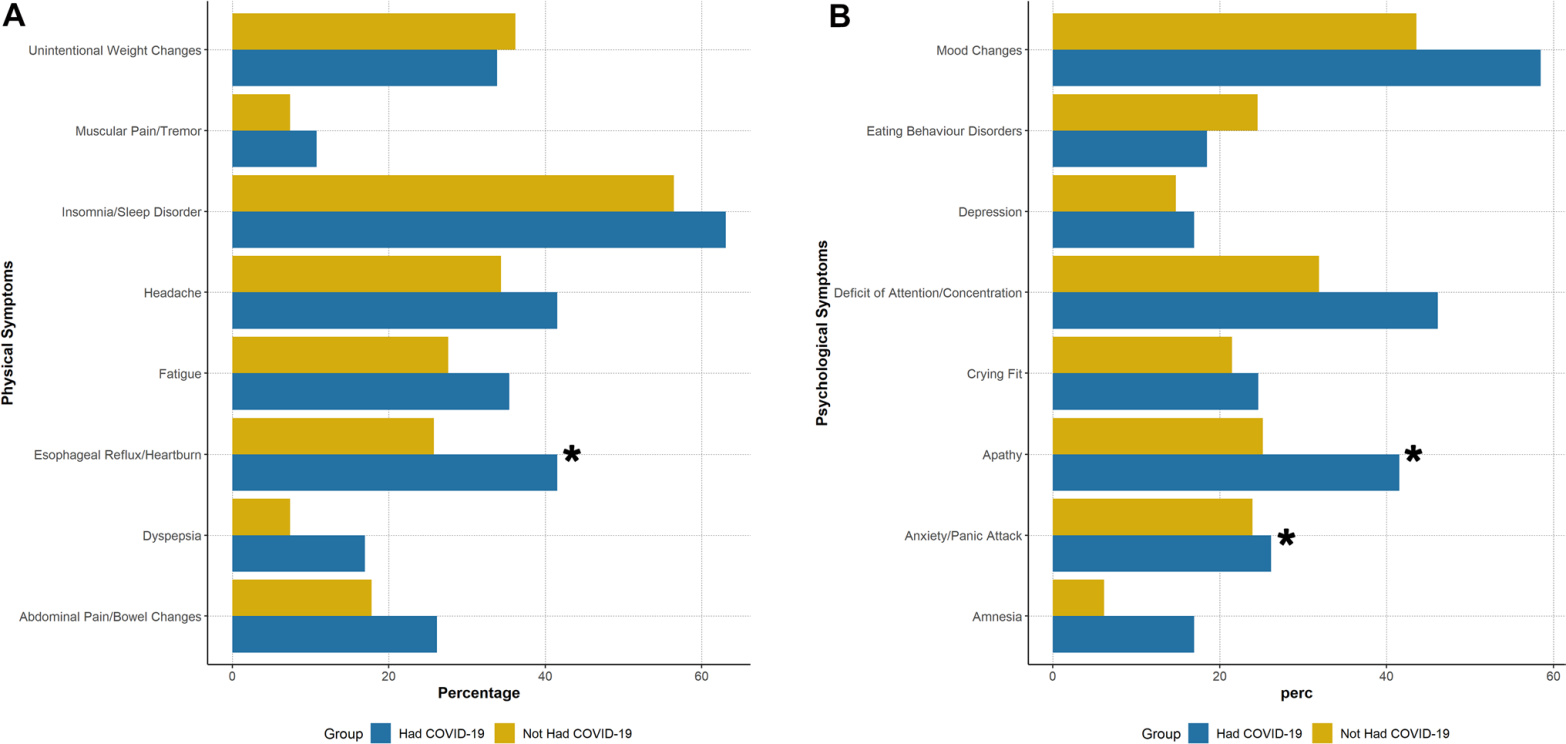
Physical and psychological symptoms reported by respondents, stratified by personal history of COVID-19. Legend: * denotes significant differences at a < 0.05 *p* levels. Panel **A**: Physical symptoms; Panel **B**: Psychological symptoms

We, therefore, compared respondents according to their job role, their involvement in COVID-19 units, and their personal history of COVID-19 infections. Table 1 and Table 2 report results for these comparisons, while graphical representations of the differences observed among the three comparisons are reported in Figs. 1, 2, 3. Additionally, we also compared respondents according to their geographical location (center north vs. center south of Italy; Supplementary Tables 1 and 2).

Compared to specialists, residents were younger (mean age 29.5 ± 2.6 vs. 42.6 ± 10.3, respectively, *p* < 0.001), and more frequently had with COVID-19 (*p* = 0.032). They were also more likely to work more than 45 hours per week (42.5% vs. 26.9, *p* = 0.020), while specialists were more frequently attending more than 4 night shifts per month (35.2% vs. 22.5%, *p* = 0.049). No significant differences emerged on self-reported perception on work changes, nevertheless specialists were more frequently reported worsening of relationship with colleagues (*p* = 0.037). Apathy and attention deficits were reported more frequently by residents compared to specialists (40.0% vs. 18.5%, *p* = 0.001, and 42.5% vs. 28.7%, *p* = 0.042, respectively). A similar trend, although non-statistically significant, was observed for crying fits, which were numerically more reported among residents (*p* = 0.072). Moreover, residents more frequently reported empty feelings at work (35.8% vs. 18.5%, *p* = 0.006), as well as a non-significant trend towards higher prevalence of amnesia, challenges in interacting with colleagues, frustration and sense of inadequacy. No statistically significant difference was observed for the physical symptoms.

When comparing those who did and did not worked in COVID-19 wards, we found that the former were more likely internal medicine specialists (*p* = 0.006) and showed a trend towards higher workload. Participants who worked in COVID-19 units more frequently reported sense of inadequacy during work shifts (58.7% vs. 34.4%, *p* = 0.018), with also trends towards lower proportion reporting amnesia (*p* = 0.082) and fear of infecting him/herself (*p* = 0.063). Consistently, those working in COVID-19 wards were less frequently reporting neutral feelings after shifts, with numerically higher proportion of respondents who suffered from both negative and positive feelings (*p* = 0.005). Finally, a numerically higher proportion of those who worked in COVID-19 wards reported difficulties in communicating worsening prognosis or death to patients or their families (*p* = 0.067).

We also compared physicians according to their personal history of COVID-19 infection. Among those infected, we found higher proportion of participants working more than 45 hours per week (46.2% vs. 30.7%, *p* = 0.040), as well as a significant higher prevalence of esophageal reflux and heartburn (41.5% vs. 25.8%, *p* = 0.029), and a trend towards higher prevalence of dyspepsia (*p* = 0.055). Apathy and amnesia were more commonly found among those who were infected with SARS-CoV-2 (*p* = 0.023 and *p* = 0.022, respectively), as well as a non-statistically significant higher prevalence of attention deficits and mood instability were reported in this group. Furthermore, physicians who experienced COVID-19 reported more frequently to have felt cynicism during work (41.5% vs. 22.1%, *p* = 0.005), as well as a trend toward lower fear of infecting themselves (*p* = 0.098) and higher prevalence of empty feelings (*p* = 0.069). They also more frequently reported to have experienced misunderstandings and arguments with familiar members (41.5% vs. 19.0%, *p* = 0.001) and sense of guilty (23.1% vs. 11.7%, *p* = 0.048).

Overall, 212 (92.3%) participants reported their geographical location; results are reported in Supplementary Tables 1 and 2. There was no significant difference between respondents from center north and center south of Italy in term of job role or involvement in COVID-19 units; however, more respondents from center north of Italy reported to have been infected with COVID-19 (34.4% vs. 12.1%, *p* = 0.002). Participants from north of Italy were more likely to work more than 45 hours per week (*p* < 0.001) while less frequently doing more than 4 night shifts per month (*p* = 0.006). On the other side, those from center south of Italy reported more frequently worsening relationship with colleagues (*p* = 0.002). Prevalence of esophageal reflux and heartburn was higher among center north-based physicians (36.4% vs. 19.0%, *p* = 0.024), who also reported higher prevalence of work-related symptoms, including feeling of sadness (42.2% vs. 25.9%, *p* = 0.042) and emptiness (33.1% vs. 17.2%, *p* = 0.035). No other statistically significant differences were observed for other symptoms, nor related to the impact of COVID-19 on the relationships with patients and physicians’ families.

## Discussion

In the present manuscript, we analysed data from a nationwide survey of Italians physicians comprehensively explore the impact of pandemic on their well-being. The most relevant findings are as follows: physicians experienced a significant amount of stress during the pandemic, as encompassed by a considerable proportion working more than 45 hours per week and/or more than four night shifts per months; this reflected into a high burden of physical and psychological symptoms reported as new-onset or worsened during the pandemic. Unsurprisingly, respondents pointed out about worsening of work organization and patient–physician relationship. Nevertheless, most physicians have witnessed a familiar impact of their work-related stress, which in turn partly influenced the self-reported work performance in a non-negligible proportion of participants. Finally, several differences were observed among different groups or participants, particularly between residents and specialists.

COVID-19 had a tremendous impact on the life of billions of people worldwide, with increased morbidity and mortality and indirect effects that are difficult to estimate in the context of the pandemic. Nonetheless, the “multidimensional” impact (i.e., encompassing the psychological and overall well-being) of COVID-19 has also been repeatedly stressed in the literature as a common detrimental effect [16–19]. HCPs are among the most affected individuals being in charge of constituting the frontline against the pandemic, overwhelmed by the enormous burden of critical patients, with little knowledge of the pathology, especially in the first phases of this outbreak [3]. Among HCPs, those working in emergency and general medicine units, largely represented by internal medicine specialists, were particularly involved in the treatment of COVID-19 patients.

The survey has been conducted in the midst of the second wave of the pandemic in Italy and explored the multifaceted effect of the COVID-19 pandemic on the well-being of a representative sample of the Italian physicians. Notably, more than half of participants were young resident physicians, offering us an unparalleled insight into a group of HCPs which is particularly prone to the effect of COVID-19, especially on psychological symptoms.

Most of the participants were directly involved in the management of COVID-19 patients, and reported a significant increase in the workload during the pandemic. Unsurprisingly, more than half of respondents remarked significant worsening of the work organization and 2 out of 3 physicians pointed out a detrimental effect of the pandemic on the patient–physician relationship; previous analysis on the doctor–patient relationship had provided mixed-evidence on the topic [20, 21], emphasizing how self-reported perception may play a role in this context.

Moreover, the present analysis showed how physicians experienced a significant burden of physical and psychological symptoms. Insomnia and sleep disturbances were among the most reported physical symptoms, along with weight changes, fatigue and gastroesophageal reflux. Nevertheless, almost half physicians experienced mood changes and 1 out of 3 reported attention difficulties as well as apathy. Taken together, these findings may stand for a more general physical, mental and emotional exhaustion, which has already been reported among HCPs during COVID-19 [22–24], and represents one of the most neglected topics of the pandemic.

Important differences were found between residents and specialists. Residents were expectedly younger, and were infected more with COVID-19. Furthermore, apathy and deficit of attentions were significantly more frequently reported among residents, and a numerically higher proportion of patients with insomnia, sleep disorders, mood swings and anxiety were found among young physicians. These findings are particularly important and may have several interpretations. Young physicians may be more prone to the physical and mental effects of COVID-19, given their shorter work experience and the abrupt increase in their clinical responsibilities and commitments. Consistently, residents more frequently reported a higher number of working hours per week, and a numerically higher prevalence of most work-related symptoms, including the sense of inadequacy. Finally, young physicians reported more frequently to have suffered from “sense of emptiness” during work, in accordance with the hypothesis of a higher risk of emotional exhaustion.

Existing evidence has already pointed out the risk of burnout in younger HCPs [25–27]. Thus, our findings are particularly important in view of this previous evidence. While further studies are clearly needed to provide definitive answers on the topic, it is conceivable that many of the known risk factors for burnout may have been particularly exacerbated during the COVID-19 pandemic. Along with the uncertainties and high level of stress experienced during the outbreak, the pandemic-related stress has greatly affected young physicians.

On the other side, although we did not find any significant difference in physical and psychological symptoms reported by physicians employed in COVID-19 units compared to those not directly involved in the treatment of COVID-19 patients, these results may have been influenced by the low number of respondents that did not work in COVID-19 units. Nevertheless, a numerically higher proportion of respondents among those working in COVID-19 wards was observed for almost all physical symptoms and most psychological findings, and particularly self-reported mood alterations and eating behaviour disorders. Similar results were observed for work-related symptoms, with COVID-19-involved HCPs reporting more frequently to have felt sense of inadequacy and a non-statistically significant higher proportion of several other symptoms. Consistently, the impact on familiar relationships resulted stronger among this group of participants, underlining the affective cost imposed by COVID-19 in HCPs, far beyond the work-related impact.

Notably, physical and psychological findings were reported in a higher proportion of physicians infected with COVID-19, including a statistically higher proportion of participants who experienced esophageal reflux, apathy and anxiety. While these effects may been related to the COVID-19 itself, it should also be noted that younger physicians were over-represented among those infected, and, therefore, these results should be interpreted in view of the aforementioned speculations and hypotheses. Finally, interesting regional differences were observed among respondents, with physicians from center—northern regions of Italy reporting a higher burden of work-related symptoms, a higher prevalence of previous infection with COVID-19 and, on the other side, a lower effect of the pandemic on their relationship with colleagues. These differences may be useful to analyse the differential impact on the pandemic according to regional differences, also considering the heterogeneous burden of the pandemic in the different regions of Italy [15].

Taken together, our results offer an important outlook on the impact of COVID-19 on the overall well-being of Italian physicians. The impact of the pandemic went far beyond the physical and mental consequences of the extremely challenging work-related experiences, also affecting the relationship with patients and their relatives, as well as the physician’s interactions with their own families. Several open questions remain, including the long-term consequences, especially on the mental health well-being of HCPs who were battered during COVID-19. A close surveillance and further studies are needed in the next years, to clarify whether these findings apply to other scenario and will reflect in higher incidence of burnout, especially among young physicians.

### Strength and limitations

The principal strength of our studies relies on the vast amount of information collected from respondents, which helped us to evaluate the multifaceted impact of COVID-19 pandemic far beyond the influence on physical and mental health, also on the relationship between physicians and patients, colleagues and families. Moreover, participants in this survey were equally distributed between specialists and residents, thus allowing to perform comparison between the two groups. This let us find several important differences which may be important in the design of further studies and preventive strategies to tackle the impact of pandemics and stressing situations on well-being of HCPs, especially the youngest ones.

Our study has also some important limitations. First, no data on the sex of the participants was collected. We decided not to collect data on sex in this survey, to ensure anonymity of the participants; as we also collected data on age, job role, workplace and region of residence. We acknowledge this as a limitation of our work, which should be addressed by further studies, also considering that previous studies reported a significant contribution of sex on the physical and mental health of health professionals in the COVID-19 pandemic [28–30]. Overall, this limitation may contribute to reduce the generalizability of our findings. Furthermore, our sample size may be not powered enough to detect significant differences between the groups compared, and particularly among those who did vs. did not work in COVID-19 units. Our analyses were not adjusted for multiple comparisons; therefore, the results should be taken as hypothesis generating. In addition, our study was conducted in a single country and most respondents were internal medicine specialists or residents, thus it may not be representative of the overall population of HCPs which has been involved during COVID-19 pandemic.

## Conclusions

In this Italian-based survey, we found a considerable impact of COVID-19 on physical and mental well-being of physicians, especially among the youngest ones. Pandemic also influenced work organizations, doctor–patient relationship and social interactions of physicians, with important differences observed between residents and specialist and those who were employed or not in COVID-19 units. Further studies are needed to evaluate the long-term consequences of the COVID-19 pandemic on the health status of HCPs.

## Supplementary Information

Below is the link to the electronic supplementary material.

Supplementary file1 (DOCX 103 KB)
