# Supplementary material for: The impact of COVID-19 pandemic on well-being of Italian physicians: a report from the Italian Society of Internal Medicine (SIMI) national survey
Source: Intern Emerg Med. 2022 Oct 1;18(1):53–65. doi: 10.1007/s11739-022-03108-4 (PMC9526381; doi:10.1007/s11739-022-03108-4)
Supplement: Supplementary file 1 — Supplementary file1 (DOCX 103 KB) [file 11739_2022_3108_MOESM1_ESM.docx]

**The impact of COVID-19 pandemic on well-being of Italian physicians: a report from the Italian Society of Internal Medicine (SIMI) survey**

**Supplementary Table 1 – Baseline characteristics and physical and mental symptoms of Respondents, stratified according to Geographical Region**

| **Variables, n (%)** | **Center North (n=154)** | **Center South (n=58)** | **P** |
| --- | --- | --- | --- |
| Age, years (Mean ± SD) | 35.5 ± 10.4 | 35.9 ± 7.9 | 0.787 |
| *Job Role* |  |  | 0.147 |
| Resident | 88 (57.1) | 26 (44.8) |  |
| Specialist (Consultant, Attending) | 66 (42.9) | 32 (55.2) |  |
| *Worked in COVID-19 wards* |  |  | 0.800 |
| Yes | 134 (87.0) | 49 (84.5) |  |
| No | 20 (13.0) | 9 (15.5) |  |
| *History of COVID-19* |  |  | 0.002 |
| Yes | 53 (34.4) | 7 (12.1) |  |
| No | 101 (65.6) | 51 (87.9) |  |
| *Medical Specialty* |  |  | 0.829 |
| Internal Medicine | 109 (70.8) | 42 (72.4) |  |
| Geriatrics | 14 (9.1) | 7 (12.1) |  |
| Emergency Medicine | 4 (2.6) | 1 (1.7) |  |
| Other | 27 (17.5) | 8 (13.1) |  |
| *Workload* |  |  |  |
| More than 45 hours per week | 65 (42.2) | 7 (12.1) | <0.001 |
| More than 4 night shifts per month | 35 (22.7) | 25 (43.1) | 0.006 |
| **Questionnaire** |  |  |  |
| *Did you notice the onset or worsening of any of these physical signs/symptoms?* | | | |
| Esophageal Reflux, Gastric Pyrosis | 56 (36.4) | 11 (19.0) | 0.024 |
| Astenia | 45 (29.2) | 18 (31.0) | 0.929 |
| Weight gain or loss | 57 (37.0) | 22 (37.9) | 1.000 |
| Headache | 51 (33.1) | 26 (44.8) | 0.155 |
| Dyspepsia | 21 (13.6) | 2 (3.4) | 0.060 |
| Abdominal pain/Bowel habit changes | 31 (20.1) | 10 (17.2) | 0.780 |
| Muscular pain/tremor | 15 (9.7) | 4 (6.9) | 0.707 |
| Insomnia/Sleep disorder | 97 (63.0) | 29 (50.0) | 0.119 |
| *Did you notice the onset or worsening of any of these other sign/symptoms?* | | | |
| Anxiety/panic attack | 39 (25.3) | 13 (22.4) | 0.795 |
| Apathy | 49 (31.8) | 17 (29.3) | 0.853 |
| Amnesia | 15 (9.7) | 5 (8.6) | 1.000 |
| Crying spells | 37 (24.0) | 9 (15.5) | 0.249 |
| Attention deficits/Difficulties in concentrating | 56 (36.4) | 21 (36.2) | 1.000 |
| Eating behaviour disorders | 39 (25.3) | 12 (20.7) | 0.601 |
| Depression | 27 (17.5) | 6 (10.3) | 0.283 |
| Mood swings | 78 (50.6) | 26 (44.8) | 0.547 |

**Supplementary Table 2 – Work-related symptoms and self-reported familiar impact in Respondents, stratified according to Geographical Region**

| **Variables, n (%)** | **Center North (n=154)** | **Center South (n=58)** | **P** |
| --- | --- | --- | --- |
| **Questionnaire** |  |  |  |
| *Do you think your work organization has worsened during COVID-19?* | | |  |
| Not Worsened/Improved | 34 (22.1) | 21 (36.2) | 0.089 |
| Slightly Worsened | 33 (21.4) | 8 (13.8) |  |
| Significantly Worsened | 87 (56.5) | 29 (50.0) |  |
| *Do you think that patient-physicians relationship has worsened during COVID-19?* | | | |
| Not Worsened/Improved | 55 (35.7) | 18 (31.0) | 0.704 |
| Slightly Worsened | 47 (30.5) | 17 (29.3) |  |
| Significantly Worsened | 52 (33.8) | 23 (39.7) |  |
| *Do you think that your relationship with patients’ relatives has worsened during COVID-19?* | | | |
| Not Worsened/Improved | 41 (26.6) | 12 (20.7) | 0.531 |
| Slightly Worsened | 45 (29.2) | 21 (36.2) |  |
| Significantly Worsened | 68 (44.2) | 25 (43.1) |  |
| *Do you think that your relationship with other colleagues has worsened during COVID-19?* | | | |
| Not Worsened/Improved | 97 (63.0) | 23 (39.7) | 0.002 |
| Slightly Worsened | 39 (25.3) | 18 (31.0) |  |
| Significantly Worsened | 18 (11.7) | 17 (29.3) |  |
| *Did you notice the onset or worsening of any of these when at work?* | | | |
| Work-related anxiety | 45 (29.2) | 13 (22.4) | 0.413 |
| Amnesia | 6 (3.9) | 4 (6.9) | 0.579 |
| Cinism | 43 (27.9) | 16 (27.6) | 1.000 |
| Attention deficits/Difficulties in concentrating | 45 (29.2) | 21 (36.2) | 0.416 |
| Challenges in interacting with colleagues | 33 (21.4) | 16 (27.6) | 0.444 |
| Frustration | 90 (58.4) | 35 (60.3) | 0.925 |
| Fear of infecting him/herself | 60 (39.0) | 19 (32.8) | 0.501 |
| Low empathy | 29 (18.8) | 10 (17.2) | 0.946 |
| Sense of inadequacy | 95 (61.7) | 28 (48.3) | 0.108 |
| Sadness | 65 (42.2) | 15 (25.9) | 0.042 |
| Empty feelings | 51 (33.1) | 10 (17.2) | 0.035 |
| Want to cry | 37 (24.0) | 8 (13.8) | 0.151 |
| Want to quit working | 62 (40.3) | 15 (25.9) | 0.075 |
| *After a shift in COVID-19 wards, your feelings are* | | | |
| Neutral | 57 (37.0) | 22 (37.9) | 0.901 |
| Negative | 69 (44.8) | 27 (46.6) |  |
| Positive | 28 (18.2) | 9 (15.5) |  |
| *During the pandemic, how many times did you think about work during your free time?* | | | |
| Never/Almost never | 3 (1.9) | 3 (5.2) | 0.429 |
| Sometimes | 22 (14.3) | 9 (15.5) |  |
| Often/Always | 129 (83.8) | 46 (79.3) |  |
| *During the COVID-19 pandemic, what issues did you experience in the patient-physician relationship?* | | | |
| Impairment of verbal communication with patients | 79 (51.3) | 28 (48.3) | 0.812 |
| Impairment of non-verbal communication with patients | 48 (31.2) | 18 (31.0) | 1.000 |
| Impairment of verbal communication with patients’ relatives | 85 (55.2) | 28 (48.3) | 0.456 |
| Difficulties in communicating worsening prognosis or death to patients or their relatives | 73 (47.4) | 30 (51.7) | 0.684 |
| Difficulties in communicating/explaining the care/treatment programme | 42 (27.3) | 15 (25.9) | 0.974 |
| Increased mistrust by patients or their relatives | 47 (30.5) | 20 (34.5) | 0.698 |
| *Do you experienced any of the following in your familiar relationship during the pandemic?* | | | |
| Apathy | 23 (14.9) | 7 (12.1) | 0.754 |
| Misunderstanding/Arguments | 45 (29.2) | 13 (22.4) | 0.413 |
| Fear of infecting family members | 51 (33.1) | 14 (24.1) | 0.273 |
| Reduced libido/sexual activity | 40 (26.0) | 10 (17.2) | 0.249 |
| Sense of guilty | 25 (16.2) | 8 (13.8) | 0.822 |
| Sense of abandonment | 18 (11.7) | 7 (12.1) | 1.000 |
| *Do you think that the changes in your familiar interactions have influenced your performance at work?* | | | |
| No | 92 (59.7) | 37 (63.8) | 0.310 |
| Only slightly influenced | 43 (27.9) | 18 (31.0) |  |
| Significantly influenced | 19 (12.3) | 3 (5.2) |  |
